# Characteristics of human adipose derived stem cells in scleroderma in comparison to sex and age matched normal controls: implications for regenerative medicine

**DOI:** 10.1186/s13287-016-0444-7

**Published:** 2017-02-07

**Authors:** Michelle Griffin, Caroline M. Ryan, Omar Pathan, David Abraham, Christopher P. Denton, Peter E. M. Butler

**Affiliations:** 1 Charles Wolfson Center for Reconstructive Surgery, Royal Free Hospital, London, UK; 2 Division of Surgery & Interventional Science, University College London, London, UK; 3 Department of Plastic Surgery, Royal Free Hospital, London, UK; 4 Center for Rheumatology, Royal Free Hospital, University College London, London, UK; 5 UCL Centre for Nanotechnology and Regenerative Medicine, Division of Surgery & Interventional Science, University College London, London, UK

**Keywords:** Adipose-derived stem cells, Systemic sclerosis, Scleroderma, Proliferation, Metabolism, Migration, Invasion, Regenerative medicine

## Abstract

**Background:**

Adipose-derived stem cells (ADSCs) are emerging as an alternative stem cell source for cell-based therapies. Recent data suggest that autologous ADSC-enriched micrografting improves the effects of facial involvement in systemic sclerosis (SSc). We have extensively characterised ADSCs from SSc patients and compared their phenotype and function to healthy age- and sex-matched control ADSCs.

**Methods:**

ADSCs were isolated and characterised from a cohort of six SSc patients (ADSC-SSc) and were compared to six healthy age- and sex-matched controls (ADSC-N). Cell surface phenotype lineage commitment was explored by flow cytometric analysis of mesenchymal and hematopoietic markers and by the capacity to differentiate to chondrogenic, osteogenic, and adipogenic lineages. Functional activities of ADSCs were assessed by biochemical and cellular assays for proliferation, metabolism, adhesion, morphology, migration, and invasion.

**Results:**

Upon characterization of ADSC-SSc, we found that there was no alteration in the phenotype or surface antigen expression compared to healthy matched control ADSCs. We found that the differentiation capacity of ADSC-SSc was equivalent to that of ADSC-N, and that ADSC-SSc did not display any morphological or adhesive abnormalities. We found that the proliferation rate and metabolic activity of ADSC-SSc was reduced (*p* < 0.01). We found that the migration and invasion capacity of ADSC-SSc was reduced (*p* < 0.01) compared to healthy matched control ADSCs.

**Conclusions:**

This study provides important findings that can differentially characterise ADSCs from SSc patients. Results indicate that the surface phenotype and differentiation capacity of ADSCs from SSc patients are identical to healthy matched ADSCs. While the findings indicate that the proliferation and migration capacity of ADSC-SSc is reduced, ADSC-SSc are capable of ex-vivo culture and expansion. These findings encourage further investigation into the understanding by which ADSCs can impact upon tissue fibrosis.

**Electronic supplementary material:**

The online version of this article (doi:10.1186/s13287-016-0444-7) contains supplementary material, which is available to authorized users.

## Background

Systemic sclerosis (SSc), also known as scleroderma, is an autoimmune connective tissue disease characterised by microvascular damage, dysregualtion of innate and adaptive immunity, and generalized fibrosis in the skin and multiple organs [1]. Skin fibrosis is the hallmark of this disease. However, the pathological changes in the affected organs, which include the lungs, gastrointestinal tract, kidneys, and heart, determine the clinical outcome. In the diffuse cutaneous disease subset, 10-year survival is 55% [2]. Fibrosis commonly affects the face and mouth, resulting in tightening of the skin, irreversible scarring, and retraction of the lips. A resultant narrowed oral aperture affects dental structure, speech, facial expression, psychological status, and quality of life [3].

Fibrosis associated with SSc is the result of excessive extracellular matrix production (notably collagens) by activated fibroblasts and myofibroblasts, although other structural cells, endothelial cells, platelets, and immune cells all contribute to the pathogenesis and progression of the disease [1]. Transforming growth factor (TGF)-β is thought to be one of the central dominant activation factors, while cytokines and autoantibodies are also believed to play a major role [4].

There is an unmet clinical need for new and innovative therapies to treat fibrotic diseases such as SSc. Current available therapies are targeted at treating the life-threatening complications that arise from organ involvement [5]. Recent clinical evidence suggests that lipotransfer of autologous adipose-derived stem cells (ADSCs) leads to regression of radiation-induced fibrosis and other fibrotic conditions, including SSc [6]. We have previously documented that autologous ADSC-enriched microfat transplantation is an effective intervention ameliorating the effects of fibrosis in facial SSc (http://meeting.aaps1921.org/abstracts/2015/7.cgi). ADSC-based therapies may offer an attractive therapeutic option to treat fibrosis in SSc and other fibrotic conditions.

Little is known about the therapeutic capacity and function of ADSC populations derived from SSc patients. Previous studies have attempted to characterise the phenotype of bone marrow-derived mesenchymal stem cells (BM-MSCs) from patients with autoimmune diseases. These reports are conflicting. Some studies report that BM-MSCs derived from patients with autoimmune diseases including rheumatoid arthritis (RA) and multiple sclerosis (MS) display an altered phenotype compared to their healthy counterparts, while others report that BM-MSCs retain their phenotypic properties [7–10]. BM-MSCs isolated from SSc patients display an altered phenotype when assessed for endothelial cell differentiation and angiogenesis [11–14]. However, our knowledge of the phenotype and properties of ADSCs in SSc patients is extremely limited.

Previous studies have shown perturbed bone marrow stem cell function in SSc and this may be central to disease development or progression in the late stage [11–14]. By demonstrating that ADSCs are functionally intact would perhaps offer the best opportunity for optimal autologous stem cell treatment. The aim of this study was to characterise ADSCs derived from abdominal donor sites in a cohort of SSc patients compared to ADSCs derived from a healthy sex- and age-matched control group. Characterisation of patient-derived ADSCs will aid our understanding of the potential advantages and likely impact of this treatment option in SSc.

## Methods

All chemicals were purchased from Sigma Aldrich unless otherwise stated.

### Donor specification

Subcutaneous lipoaspirate from the abdomen was obtained from six females with systemic SSc. The age range of the patients was 28–40 years (mean age 34 ± 6 years). An age-matched female cohort of six patients undergoing lipotransfer from the abdomen was used as the healthy matched controls (average age 34 ± 7 years). The medications prescribed to each of the participants in this study are detailed in Additional file 1: Table S1.

### Isolation of ADSCs

ADSCs were isolated according to a modified method as described by Naderi et al. [15]. In brief, after the removal of fibrous tissue and visible blood vessels, samples were cut into small pieces (3 mm^3^) and digested in Dulbecco’s modified Eagle’s medium/Nutrient Mixture F-12 Ham (DMEM/F12) containing 300 U/ml crude collagenase II (Invitrogen, Life Technologies Ltd, Paisley, UK) for 30 min in an incubator (37 °C, 5% CO_2_). The dispersed material was filtered successively through 40- and 70-μm cell strainers (BD Biosciences, Oxford, UK). After centrifugation (290 × *g*, 5 min), the ADSC-rich cell preparation formed a pellet at the bottom of the tube. After red cell lysis the supernatant was removed and the pellet re-suspended prior to being expanded in culture.

### Culture of human ADSCs

Cells were maintained in culture in DMEM/F12 for four passages at 37 °C in a humidified atmosphere of 5% CO_2_. At each subsequent passage, cells were seeded to sub-confluence in 75 cm^2^ culture flasks at a seeding density of 0.4 × 10^3^ cells/cm^2^ (3 × 10^4^/flask) for 7–10 days. When the cells reached approximately 80% confluence, subculture was performed through trypsinization. The cell suspension was centrifuged at 290 × *g* for 5 min, the pellet was resuspended, and cells were seeded at 3 × 10^4^ per 75 cm^2^ flask.

### Cell proliferation and metabolism

Cell metabolism and proliferation was assessed by alamar blue and DNA assay, respectively. The commercially available assay Alamar blue™ (Life Technologies, UK) was used to assess viability and metabolism. The ADSCs were seeded in six-well plates at a seeding density of 1 × 10^3^/cm^2^ (1 × 10^4^ per well) to assess proliferation and metabolism at different time points including 1, 3, 7, and 14 days. Alamar blue assay was then performed as per the manufacturer’s instructions. Briefly, after 4 h of incubation with alamar blue dye, 100 μl of media was place into 96-well plates and fluorescence was measured at excitation and emission wavelength of 530 and 620 nm using Fluoroskan Ascent FL (Thermo Labsystems, UK). To assess ADSC proliferation a Fluorescence Hoechst DNA Quantification Kit was utilised to quantify the DNA content (Sigma, UK). The assay was performed using the standardised manufacturer’s protocol. The fluorescence was measured with excitation set at 360 nm and emission at 460 nm using Fluoroskan Ascent FL (Thermo Labsystems) (*n* = 6).

### Flow cytometric analysis

Passage (P)2 ADSCs were used for immunophenotopic characterisation using flow cytometry. ADSCs were stained with antibodies for different CD antigens and HLA-DR (Macs Quant Mitenyl Biotec): (antibodies were not diluted) CD14-Viogreen, Anti-HLA-DR-PerCP-Vio700, CD34-Vioblue, CD19-APC-Vio770, CD73-PE, CD90-FITC, and CD105-APC. The antibody isotope was used as controls as per the manufacturer’s instructions (Mitenyl Biotec). Data acquisition was performed with MacsQuant Analyser 10 and data were analysed using Macs quant analysis software (*n* = 6).

### Adipose stem cell differentiation

Adipose stem cells were differentiated to adipogenic, chondrogenic, and osteogenic lineages according to Guasti et al. [16].

#### Adipogenic differentiation: quantification

ADSCs were cultured to confluence for 3 days and then incubated with a medium containing DMEM, 10% fetal bovine serum (FBS), 10 ng/ml insulin, 500 mM 3-isobutyl-1-methylxanthine, 1 mM dexamethasone, and 1 mM rosiglitazone. After 3 weeks, cells were fixed with 4% paraformaldehyde (PFA), washed with 60% isopropanol for 5 min, and stained with Oil Red O (0.25% wt/vol) for 10 min. After staining, cells were washed several times with H_2_O. For quantification, the dye was extracted with 100% isopropanol for 30 min at room temperature. Optical density at 495 nm was determined with a plate reader (Bio-Rad, Hercules, CA; http://www.bio-rad.com). Fold-change was calculated by using untreated controls as a reference (*n* = 6).

#### Chondrogenic differentiation: quantification

Confluent ADSCs were incubated in a medium containing DMEM, 10% FBS, 0.1 μM dexamethasone, 10 ng/ml TGF-β1 (R&D Systems, UK), insulin-transferrin-selenium (ITS) (Life Technologies), and 50 μg/ml ascorbate. After 3 weeks, cells were fixed in 4% PFA, rinsed with 0.1 N HCl for 5 min and stained with Alcian Blue (1% in 0.1 N HCl). For quantification, the dye was extracted with 6 M guanidine hydrochloride overnight at room temperature, and optical density at 595 nm was determined. Fold-change was calculated by using untreated controls as a reference (*n* = 6).

#### Osteogenic differentiation: quantification

Confluent cells were incubated in a medium containing DMEM, 10% FBS, 0.1 μM dexamethasone, 100 μg/ml ascorbate, and 10 mM β-glycerophosphate. After 3 weeks, cells were fixed in ice-cold 70% ethanol for 1 h, washed with H_2_O, and stained with 1% Alizarin Red. For quantification, stained cells were incubated with 10% acetic acid for 30 min at room temperature, scraped, transferred to 1.5-ml vials, and heated at 85 °C for 10 min. Debris was eliminated by centrifugation and the optical density of the supernatants was measured at 405 nm. Fold-change was calculated by using the untreated controls as a reference (*n* = 6).

### Reverse transcription-polymerase chain reaction and quantitative real-time polymerase chain reaction

RNA was extracted from cells using Trizol (Life Technologies) according to the manufacturer’s protocol. All cells were examined after 3 weeks of culture as described above. RNA was isolated by phenol extraction and ethanol precipitation. One microgram of RNA was then reverse transcribed with Moloney murine leukemia virus reverse transcriptase (Promega, Madison, WI; http://www.promega.com). The resultant cDNA was amplified using GoTaq (Promega; Applied Biosystems, Foster City, CA; http://www.appliedbiosystems.com). mRNA was quantified by real-time quantitative polymerase chain reaction with the Prism 7500 sequence detection system (Applied Biosystems) and the QuantiTect SYBR Green PCR Kit (Qiagen, Hilden, Germany; http://www1.qiagen.com) following the manufacturers’ instructions. Gene expression data were normalized using *GAPDH* as a reference (*n* = 3). The osteogenic genes collagen type 1 (*ColI*), alkaline phosphatase (*ALP*), and osteocalcin (*OC*) were evaluated. The chondrogenic genes aggrecan (*Agg*) and collagen type II (*ColII*) were examined. The adipogenic genes fatty acid binding protein 4 (*FABP4*), peroxisome proliferator-activated receptor gamma (*PPAR*γ), CCAAT/enhancer binding protein alpha (*C/EBBP*α), and lipoprotein lipase (*LPL*) were investigated.

### Cell morphology and adhesion

To study ADSC morphology between P1 and P4, immunocytochemistry staining was carried out following 24 h of culture. Firstly, the media was removed from the 24 wells and the cells were washed with phosphate-buffered saline (PBS) three times. Following this, the cells were fixed with 4% (w/v) PFA in pre-warmed PBS at 37 °C for 10–15 min. Cells were then washed with 0.1% Tween 20 three times, followed by addition of 0.1% triton X100 for 5 min to improve permeability. The cells were then stained with Rhodamine Phalloidin dye at 1:40 (diluted in 1000 μl methanol) in PBS for 40 min. Cells were then washed three times and mounted onto slides with DAPI to stain the nuclei. The cells were visualized under fluorescence microscopy (EVOS) at 40× magnification (*n* = 6). Cell morphology was carried out using ImageJ software; five random fields of view of four cells were identified and assessed (*n* = 20). To assess ADSC adhesion, the number of cells attached was assessed over a 24-h time period using a Fluroscence Hoechst DNA Quantification Kit to quantify DNA content (Sigma, Gillingham, UK). The assay was performed as per the manufacturer’s instructions. The fluorescence was measured with excitation set at 360 nm and emission at 460 nm using Fluoroskan Ascent FL (Thermo Labsystems).

### Migration assay

In order to determine the migratory capacity of ADSCs, the QCM 24-Well Colorometric Cell migration Assay (Merk Millipore) was performed as per the manufacturer’s protocol. Briefly, ADSCs were seeded in expansion medium without FBS at 10,000 cells per insert. The lower well contained expansion medium with 10% FBS. The plate was then incubated at 37 °C at 5% CO_2_ for 24 h. After 24 h, the inserts were transferred into new wells containing 400 μl cell stain for 20 min. The inserts were then washed with PBS and the non-migrated cells removed from the interior with cotton wool swabs. The dried inserts were then transferred into 200 μl extraction buffer for 15 min. The optical density was then measured at 560 nm (*n* = 6).

### Invasion assay

The invasion capacities of ADSCs were assessed with a Cell Invasion Assay (QCM ECMatrix Cell Invasion Assay, Merck Millipore). ADSCs were seeded in expansion medium without FBS at 10,000 cells per insert. The lower well contained expansion medium with 10% FBS. The plate was then incubated at 37 °C at 5% CO_2_ for 24 h. After 24 h, the medium was removed and the non-invading cells on the interior of the inserts were removed with cotton wool swabs. The inserts were then transferred into 500 μl staining solution for 30 min. Inserts were then washed with PBS and transferred into 200 μl extraction buffer. The optical density of 200 μl extracted dye was then measured at 480/520 nm (*n* = 6).

### Statistical analysis

Inter-treatment comparisons were analysed statistically using one-way analysis of variance (ANOVA) with Tukey HSD post-hoc analysis (Prism6 Software). The average and standard deviation (SD) was calculated. Significance was described as *p* < 0.05. Graphpad was used for graphically representing data.

## Results

### Phenotypic characterisation of ADSCs isolated from SSc patients

**Fig. 1:**
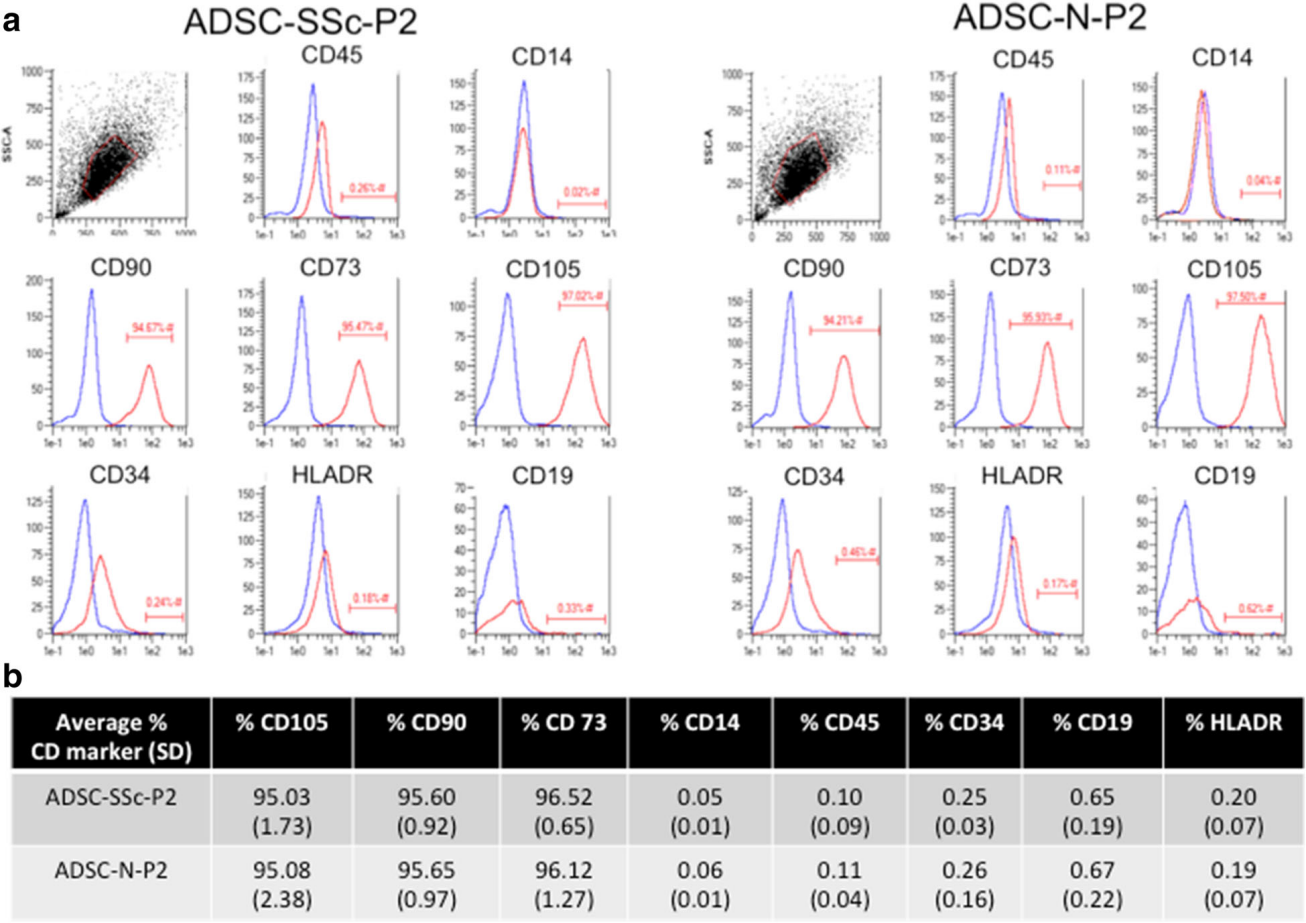
Flow cytometric analysis of adipose-derived stem cells from systemic sclerosis patients (*ADSC-SSc*) and from a healthy age- and sex-matched control cohort (*ADSC-N*). ADSCs from SSc patients are phenotypically identical to healthy control ADSCs. **a** Example of the flow cytometric dot plot, histogram analysis, and the gating used to obtain percent expression on the surface of ADSC-SSc and ADSC-N at passage 2 (*P2*). **b** Summary table displaying the average percentage of antigens for each FACS marker expressed on the surface of the cells. FACS analysis was performed on ADSC-SSc and ADSC-N at P2. FACS analysis was performed for mesenchymal markers CD105, CD90, CD73, and hematopoietic markers CD14, CD45, CD34, CD19, and HLA-DR. *SD* standard deviation

Adipose-derived stem cells (ADSCs) were isolated following liposuction from a cohort of six female SSc patients (ADSC-SSc). ADSCs were also isolated from a healthy sex- and age-matched control group of six that were undergoing lipotransfer from the abdomen (ADSC-N). Flow cytometric analysis of mesenchymal and hematopoietic markers was carried out to characterise the phenotype of ADSC-SSc. Fluorescence-activated cell sorting (FACS) analysis was performed on ADSC-SSc and ADSC-N at P2 (Fig. 1a). ADSC-SSc meet the classification criteria for mesenchymal stem cells according to the criteria of the International Society for Cellular Therapy (ISCT) and the International Federation for Adipose Therapeutics and Science (IFATS) [17]. ADSC-SSc were positive for CD105, CD90, and CD73, and negative for hematopoietic markers CD45, CD34, CD19, CD14, and HLA-DR (Fig. 1b). Using this defined set of cell surface markers, ADSCs from SSc patients were phenotypically identical to healthy control ADSCs (Fig. 1b). The FACS profiles of all of the participants in the study are detailed in Additional file 2: Table S2.

### Comparison of ADSC differentiation capacity from SSc patients and healthy controls

**Fig. 2:**
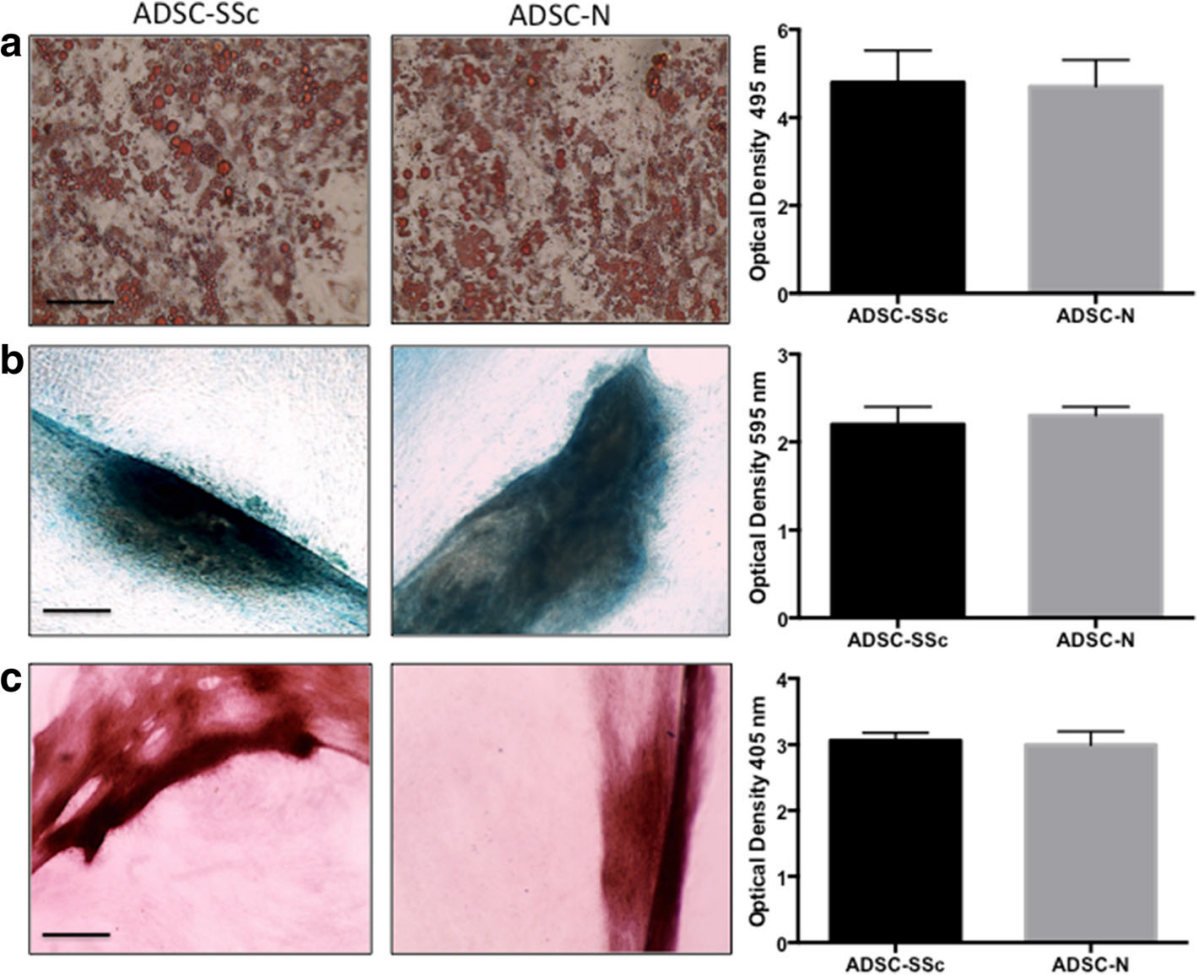
Differentiation capacity of ADSCs from SSc patients. ADSCs from SSc patients have comparable differentiation capacity to healthy control ADSCs. **a** Adipogenic differentiation of adipose-derived stem cells from SSc patients (*ADSC-SSc*; *left image*) and ADSCs from healthy controls (*ADSC-N*; *right image*) was assessed by Oil Red O staining. Positive Oil Red O staining and fat globules were observed in both ADSC-SSc and ADSC-N at 3 weeks. Quantitative analysis of Oil Red O staining at optical density of 495 nm (*graph on right*) determined that there was no difference in the adipogenic differentiation capacity of ADSC-SSc compared to ADSC-N (10× magnification). **b** Chondrogenic differentiation of ADSC-SSc (*left image*) and ADSC-N (*right image*) was assessed by Alcian Blue dye. Positive Alcian Blue staining for proteoglycans was observed in both ADSC-SSc and ADSC-N at 3 weeks. Quantitative analysis of Alcian blue staining at optical density of 595 nm (*graph on right*) determined that there was no difference in the chondrogenic differentiation capacity of ADSC-SSc compared to ADSC-N (10× magnification). **c** Osteogenic differentiation of ADSC-SSc (*left image*) and ADSC-N (*right image*) was assessed by Alizarin Red dye. Positive Alizarin red staining for calcium was observed in both ADSC-SSc and ADSC-N at 3 weeks. Quantitative analysis of Alizarin staining at optical density of 405 nm (*graph on right*) determined that there was no difference in the osteogenic differentiation capacity of ADSC-SSc compared to ADSC-N (*n* = 3) (10× magnification)

**Fig. 3:**
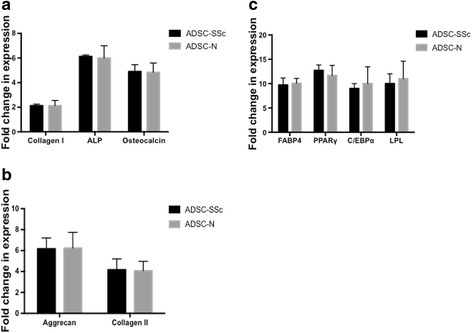
Quantitative RT-PCR analysis of osteogenic-, chondrogenic-, and adipogenic-specific genes expressed in ADSCs from SSc patients. Adipose-derived stem cells from systemic sclerosis patients (*ADSC-SSc*) have a similar gene expression profile following differentiation when compared to healthy control ADSCs (*ADSC-N*). **a** Fold-change in expression of the osteogenic genes collagen type 1 (*ColI*), alkaline phosphatase (*ALP*), and osteocalcin (*OC*) in ADSC-SSc and ADSC-N. **b** Fold-change in gene expression of the chondrogenic genes aggrecan (*Agg*) and collagen type II (*ColII*) in ADSC-SSc and ADSC-N. **c** Fold-change in gene expression of the adipogenic genes fatty acid binding protein 4 (*FABP4*), peroxisome proliferator-activated receptor gamma (*PPAR*γ), CCAAT/enhancer binding protein alpha (*C/EBBP*α), and lipoprotein lipase (*LPL*) in ADSC-SSc and ADSC-N. Gene expression profiling was carried out at 3 weeks following differentiation. Fold change was normalised to *GAPDH* (*n*=3)

To further characterise ADSCs from SSc patients, the capacity of ADSC-SSc to differentiate to adipogenic, chondrogenic, and osteogenic lineages was assessed. The differentiation potential of ADSC-SSc was compared to that of ADSC-N over a 3-week culture period. Supporting previous work [18], we found that ADSCs from SSc patients exhibited comparable differentiation capacity to ADSCs from healthy donors (Fig. 2a–c). We found no statistical difference in osteogenic, adipogenic, or chondrogenic lineages when assessed by Alizarin Red, Oil Red O and Alcian Blue, respectively (Fig. 2a–c). The capacity of ADSC-SSc to differentiate to the osteogenic lineage was further confirmed by gene expression analysis. Significantly, although we found profound increases in the expression of lineage-specific genes upon differentiation, we found no difference in the expression profile of the osteogenic genes *ColI*, *ALP*, and *OC* at day 21 in ADSC-SSc compared to ADSC-N (Fig. 3a). To confirm differentiation of ADSC-SSc to the chondrogenic lineage, the expression profile of chondrogenic genes was evaluated. Again, although we found large changes in the expression of the chondrogenic genes *Agg* and *ColII* during cell differentiation, we found no difference in the expression profile of *Agg* or *ColII* in ADSC-SSc compared to ADSC-N at day 21 (Fig. 3b). We also found no difference in the expression profile of the adipogenic genes *FABP4*, *PPAR*γ, *C/EBP*α, and lipoprotein lipase (*LPL*) in ADSC-SSc compared to ADSC-N after 21 days (Fig. 3c).

### Comparison of ADSC morphology and adhesion properties from SSc patients and healthy controls

**Fig. 4:**
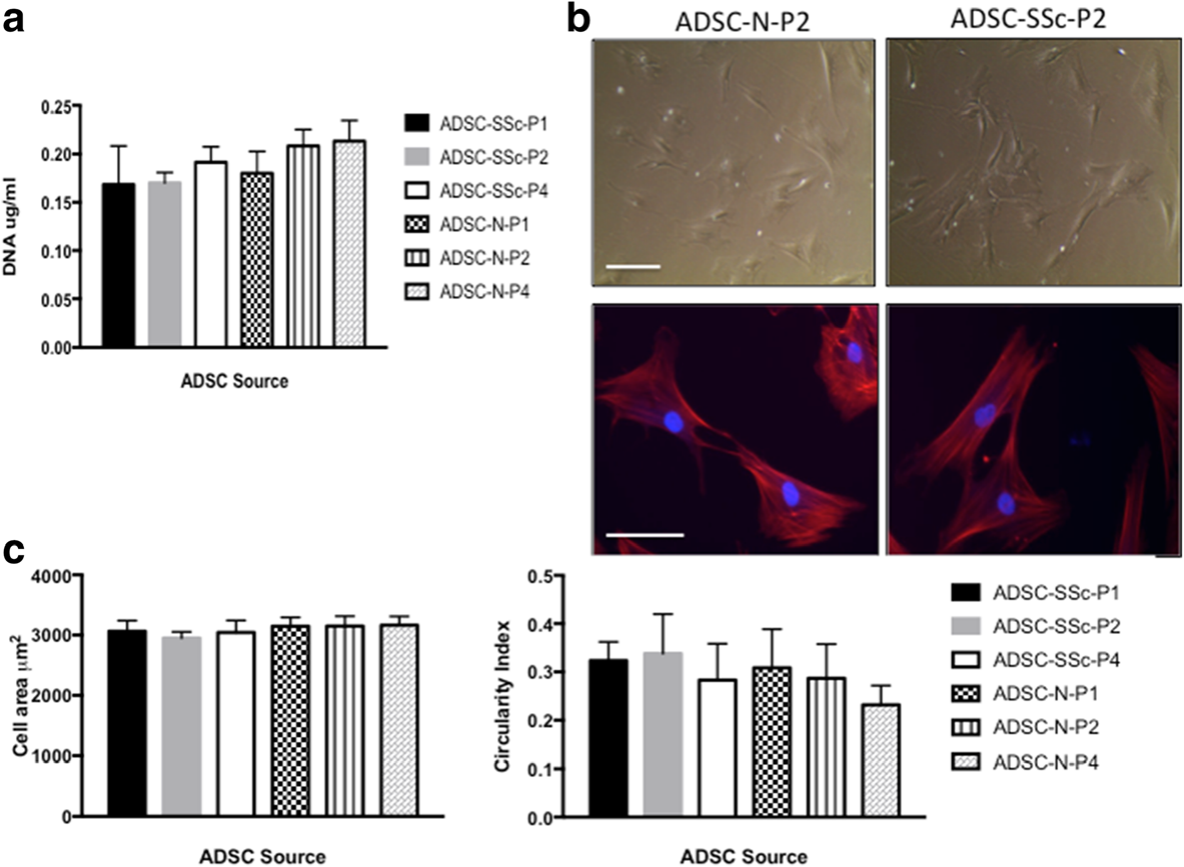
Analysis of the morphology and adhesion properties of ADSCs from SSc patients. There was no alteration in the adhesive properties or the morphology of adipose-derived stem cells from systemic sclerosis patients (*ADSC-SSc*) compared to healthy control ADSCs (*ADSC-N*). **a** DNA quantification assay was carried out to determine the number of cells adhering to tissue culture plastic 24 h after seeding. Adhesion of ADSCs was assessed at passage 1 (*P1*) to P4. There was no statistical difference in the number of cells that adhered between ADSC-SSc and ADSC-N (*n* = 6). **b** Representative bright field images of ADSC-N at passage 2 (*top left panel*) and SSc-ADSC s at passage 2 (*top right panel*) (10× magnification). Representative images of Rhodamine Phalloidin staining of F-actin in ADSC-N at passage 2 (*bottom left panel*) and ADSC-SSc at passage 2 (*bottom right panel*) (40× magnification). Cells were counter-stained with DAPI to visualize DNA. *Scale bars* = 50 μm. **c** Cell area (*left graph*) and circularity index (*right graph*) of ADSC-SSc and ADSC-N at P1 to P4 was assessed 24 h after seeding using image J software (*n* = 20). Statistical significance was calculated using unpaired *t* test

To further investigate the properties of ADSCs from SSc patients, the morphology and adhesion of ADSC-SSc were compared to healthy control ADSCs. Adhesion properties play an important role in cell-cell and cell-matrix interactions and are a vital component in aiding immunomodulation and angiogenesis [19]. We found that there was no statistical difference in the adhesive properties of ADSCs derived from SSc patients and ADSCs from healthy controls (Fig 4a). The morphology of actively proliferating ADSC-SSc was analysed at P2. Both ADSC-N and ADSC-SSc displayed a typical fibroblast-like, spindle-shaped morphology that is characteristic of ADSCs (Fig. 4b). We did not observe any morphological abnormalities such as enlargement, granulation, or vacuoles in ADSC-SSc that would indicate senescence or apoptosis [20]. ADSCs were cultured to confluence and up to P4 without any apparent morphological abnormalities (data not shown). A more detailed investigation of F-actin distribution and bundling confirmed that ADSC-SSc were morphologically identical to healthy control ADSCs (Fig 4b). There was no statistical difference in the spread or size of the ADSC-SSc at P1 to P4 compared to ADSC-N when measured for circularity index and cell area (μm^2^) (Fig. 4c).

### Comparison of ADSC proliferation and metabolism from SSc patients and healthy controls

**Fig. 5:**
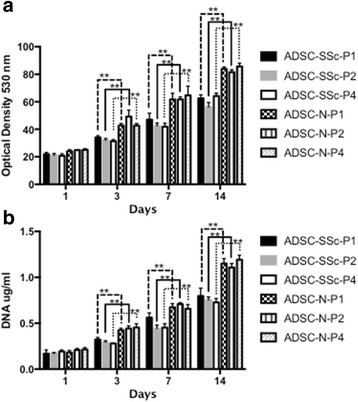
Analysis of the proliferative and metabolic properties of ADSCs derived from SSc patients. Proliferation and metabolism of adipose-derived stem cells from systemic sclerosis patients (*ADSC-SSc*) was decreased over 14 days compared to healthy control ADSCs (*ADSC-N*). **a** Cell metabolism was assessed by the Alamar blue assay on days 1, 3, 7, and 14. Cells were assessed at passage 1 (*P1*) to P4. ADSC-SSc displayed significantly less metabolic activity on days 3, 7, and 14 at all passages assessed (*p* < 0.01) (*n* = 6). **b** Cell proliferation was assessed by DNA quantification on days 1, 3, 7, and 14. Cells were assessed at P1 to P4. ADSC-SSc were significantly less proliferative on days 3, 7, and 14 at all passages assessed (*p* < 0.01) (*n* = 6). **p < 0.01

The proliferative and metabolic properties of ADSC-SSc were compared to control ADSCs. In contrast to a previous report [18], we found the proliferation rate of ADSC-SSc to be significantly reduced over 14 days compared to control ADSCs (*p* < 0.01) (Fig. 5b). A decreased proliferation rate was detected in ADSC-SSc at all passages from 1 to 4. We also found that there was significantly reduced metabolic activity in ADSC-SSc over 14 days at all passages from 1 to 4 compared to ADSC-N (*p* < 0.01) (Fig. 5a).

### Comparison of ADSC migration and invasion from SSc patients and healthy controls

**Fig. 6:**
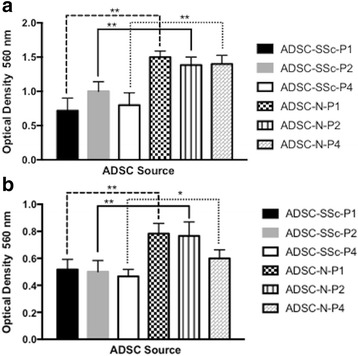
Analysis of the migration and invasion potential of ADSCs derived from SSc patients. Migration and invasion potential of adipose-derived stem cells from systemic sclerosis patients (*ADSC-SSc*) was reduced compared to healthy control ADSCs (*ADSC-N*) at all passages examined. **a** Cell migration was assessed by QCM 24-Well Colorometric Cell migration Assay. Cells were assessed at passage 1 (*P1*) to P4. Migration was assessed at 24 h. Migration of ADSC-SSc was significantly reduced at P1, P2, and P4 compared to healthy control ADSCs (*p* < 0.01) (*n* = 6). **b** Cell invasion was assessed by QCM ECMatrix Cell Invasion Assay. Cells were assessed at P1 to P4. Invasion was assessed at 24 h. Invasion of ADSC-SSc was significantly reduced at P1, P2, and P4 compared to healthy control ADSCs (*p* < 0.015) (*n* = 6). *p <0.05, **p < 0.01

We found that migration of ADSC-SSc was significantly reduced compared to control ADSCs (Fig. 6a). Migration was assessed at 24 h in response to culture medium containing the non-specific chemoattractant, fetal bovine serum (FBS). Migration of ADSC-SSc was significantly reduced at all passages (P1–P4) compared to control ADSCs (*p* < 0.01) (Fig. 6a). We also found that invasion of ADSC-SSc was significantly reduced compared to control ADSCs (Fig. 6b). Invasion was assessed by ECMatrix Cell Invasion Assay at 24 h. Invasion of ADSC-SSc was significantly reduced at all passages (P1–P4) compared to control ADSCs (*p* < 0.01) (Fig. 6b).

## Discussion

Systemic sclerosis (SSc) is characterised by abnormalities in the cutaneous, subcutaneous, and internal organ connective tissue leading to scarring and fibrosis of affected structures and also to localized diminution of subcutaneous adipose tissue in affected sites [1–4]. The disease typically progresses over time, and distinct clinical and pathological phases can be identified in many cases. It can be differentiated into limited and diffuse subsets based upon the extent and severity of skin thickening. In early-stage disease there is marked fibrosis and thickening of the skin but, at later stages, the skin may thin and become atrophic. These changes are especially marked in the forearms and hands and the face, areas that are almost always affected in both SSc subsets [21, 22]. The combination of thickening and hardening of the skin and tethering to subcutaneous structures together with loss of fat and muscle affects function and appearance. The facial involvement is often severe and has a major impact on quality of life and also function by reducing the oral aperture and reducing lip bulk and mobility [3]. Emerging data support a role for adipocyte transdifferentiation in SSc and it seems that lipoatrophy in the affected sites may in part be due to loss of adipocytes and defective stem cell function [23, 24]. Thus, it is an attractive treatment intervention to transfer fat-derived cells from proximal sites such as the abdominal wall into areas severely affected in SSc to help regenerate the adipose tissue and potentially restore connective tissue homeostasis. This is likely to have a much more profound effect if a population of stem cells can be introduced that persists and helps to remodel local connective tissue.

However, it is critical that autologous fat-derived cells maintain their normal cellular function and, in particular, that the stem cell phenotype and potential is maintained. The purpose of the present study was to assess whether ADSCs from abdominal sites in SSc are similar to those from matched healthy donors. This is important to permit autologous fat transfer procedures and provide a plausible mechanism for effects that appear to be substantial in some cases and associated with fat regeneration, improved skin flexibility, and reduced fibrosis together with better function of associated structures.

We have previously documented that adipose tissue lipotransfer significantly improves the effect of fibrosis and mouth function in facial SSc [6]. The effector cell or mechanism that is responsible for the perceived reversal of fibrosis is not yet known. Adipose-derived stem cells (ADSCs) are easily derived from adipose tissue [25]. The immunomodulatory and angiogenic effects of ADSCs are well documented, particularly in ischaemic diseases [26, 27]. It has also been suggested that ADSCs have anti-fibrotic properties through secretion of anti-fibrotic factors such as interferon (IFN)γ and matrix metalloproteinases and by decreasing pro-fibrotic factors such as TGF-β [28–31]. However, little is known about the therapeutic potential of ADSCs from SSc patients. The present study aimed to isolate, culture, and characterise ADSCs from a cohort of SSc patients (ADSC-SSc) that were undergoing lipotransfer treatment for the effects of facial SSc. The phenotype and potential of ADSC-SSc was compared to ADSC-N that were isolated from a cohort of healthy sex- and age-matched controls. We found no difference in the phenotype or tri-lineage potential of ADSC-SSc compared to ADSC-N. We did, however, find differences in the properties of ADSC-SSc compared to ADSC-N.

Adipose-derived stem cells display remarkable plasticity and undergo profound changes in phenotype during differentiation and exposure to environmental cues [32]. We found that the phenotype and surface antigen expression of patient-derived ADSC-SSc was not altered compared to ADSC-N (Fig 1a and b). Supporting this, we found that the differentiation capacity of ADSC-SSc was unchanged (Figs. 2 and 3). To date, only one study has characterised ADSCs from SSc patients [18]. Scuderi et al. similarly found that ADSCs from SSc patients were capable of differentiating towards adipogenic, osteogenic, and chondrogenic lineages [18].

Previous studies have reported that bone marrow stem cell populations from SSc patients may display early senescence [11, 13]. We did not find evidence of senescence in our abdominal-derived ADSCs. We did not detect any morphological abnormalities such as enlargement or cytoplasmic granules or vacuoles (Fig. 4b), no alteration in their adhesive properties (Fig. 4a), and, significantly, no difference in their differentiation capacity (Figs. 2 and 3), all of which would indicate a senescent phenotype [20]. Our findings provide evidence that ADSC-SSc are phenotypically identical to healthy matched ADSCs and can be cultured ex vivo in sufficient numbers for autologous therapeutic options. This difference may reflect the origin of the stem cells. It seems likely that peripheral blood or bone marrow reflect altered systemic processes in an immune-mediated disease that are not a feature of adipose-defined cells. In addition, the ability to harvest from sites that are less affected in SSc may be a key factor.

While our findings report that the phenotype and tri-lineage potential of ADSCs from SSc patients was maintained, we found that the proliferation rate and metabolic activity of ADSC-SSc was decreased (Fig. 5). This is in contrast to the previous report from Scuderi et al. [18]. Additionally, we found that the migration and invasion potential of ADSCs from SSc patients was reduced (Fig. 6a and b). Our study differed in two ways. Firstly, the patient group used in our study was a uniform patient cohort. All of the patients presented with diffuse cutaneous systemic sclerosis involving acute fibrosis of the skin and organ involvement. Secondly, ADSC-SSc in our study were compared to ADSCs from a cohort of healthy age- and sex-matched controls. It may be postulated that the severity and systemic nature of the disease presented in this patient cohort affects some of the properties of ADSC-SSc. The ability of endogenous stem cells to migrate and invade is central to their repair response [33]. It is not yet known whether a reduced capacity of endogenous SSc-ADSC populations to migrate impacts the pathology or progression of SSc. Local transplantation of autologous ADSC-SSc creates depots of cells, reducing the need to migrate and home through a compromised vascular system. Local transplantation also facilitates cell-cell contact and paracrine signalling that are essential for immunomodulation and an angiogenic response [34–36]. Our previous study demonstrates that local transplantation of autologous ADSC-SSc by lipotransfer induces a therapeutic response [6], while several other studies have reported that SSc-BM-MSCs maintain some of their immunosuppressive and angiogenic properties [9, 13, 37]. This supports the use of ADSC-SSc as an autologous therapy despite any disease-induced alteration of cellular properties.

This study assessed the ability of ADSC-SSc to be expanded in culture for therapeutic purposes. Similarly, we evaluated the phenotype and properties of ADSC-SSc in vitro at P2 to P4. Other groups have also characterised ADSCs at P2 and P3 [18]. Prior to P2, ADSC preparations are not a homogenous population and display a dynamic phenotype that changes during culture [38]. Depending on culture conditions, ADSCs in P2 or P3 are morphologically a homogenous population of fibroblastoid cells and, due to the artificial environment, will lose the expression of certain surface markers including CD34 that is used to identify ADSCs in situ and after a short culture time [38]. While it is important to characterise these cells as a homogeneous population, as we have done in this study, it will also be important to characterise the phenotype and properties of ADSC-SSc at P0 to identify any differences in surface marker expression prior to expansion in vitro. Quantification of SSc-ADSC yield at P0 will also provide important information that will aid in the enhancement of autologous ADSC therapy for systemic sclerosis patients. Future work will focus on evaluating the function of ADSC-SSc compared to ADSC-N. Secretome analysis and patient-matched co-culture studies will provide a plausible mechanism for the anti-fibrotic and restorative effects of autologous lipotransfer in SSc patients.

Several studies have shown that stem cells, both adipose- and bone marrow-derived, may allow for the reversal of fibrosis. Adipose tissue is an attractive stem cell source due to a higher yield compared to bone marrow, greater abundance, and involving a less invasive and painful harvesting procedure [21, 39, 40]. ADSCs have demonstrated superior therapeutic potential to BM-MSCs [41, 42]. Their immunomodulatory function and angiogenic properties, as well as promising clinical effects, suggests that ADSCs may offer a treatment option for several types of fibrotic diseases [42–44].

## Conclusions

Our findings confirm that ADSCs from the abdomen of SSc patients are phenotypically indistinguishable from similar cells isolated from healthy controls and can be expanded in culture for autologous therapeutic options. These findings support the feasibility of treatment approaches that transfer these ADSCs into affected sites in SSc and provide insight into potential mechanisms whereby local anti-fibrotic effects and disease modification may occur.

## Additional files

Additional file 1: Table S1. Table detailing the medications prescribed to each of the participants in this study. (DOCX 58 kb)
Additional file 2: Table S2. Table detailing the FACS profile of ADSCs of each of the participants in this study. (DOCX 54 kb)

## Abbreviations

ADSC: Adipose-derived stem cell
ADSC-N: Adipose stem cell derived from healthy matched control
ADSC-SSc: Adipose stem cell derived from systemic sclerosis patients
Agg: Aggrecan
ALP: Alkaline phosphatase
BM-MSC: Bone marrow-derived mesenchymal stem cell
C/EBPα: CCAAT/enhancer binding protein alpha
ColI: Collagen type I
ColII: Collagen type II
DMEM/F12: Dulbecco’s modified Eagle’s medium F-12 Ham
FABP4: Fatty acid binding protein 4
FACS: Fluorescence-activated cell sorting
FBS: Fetal bovine serum
IFATS: International Federation for Adipose Therapeutics and Science
ISCT: International Society for Cellular Therapy
ITS: Insulin-transferrin-selenium
LPL: Lipoprotein lipase
MS: Multiple sclerosis
OC: Osteocalcin
P: Passage
PBS: Phosphate-buffered saline
PFA: Paraformaldehyde
PPARγ: Peroxisome proliferator-activated receptor gamma
RA: Rheumatoid arthritis
SSc: Systemic sclerosis/scleroderma
ADSC-SSc: Adipose stem cell derived from systemic sclerosis patients
TGF: Transforming growth factor
