# Supplementary material for: Characteristics of human adipose derived stem cells in scleroderma in comparison to sex and age matched normal controls: implications for regenerative medicine
Source: Stem Cell Res Ther. 2017 Feb 7;8:23. doi: 10.1186/s13287-016-0444-7 (PMC5297142; doi:10.1186/s13287-016-0444-7)
Supplement: Additional file 2: Table S2. — Table detailing the FACS profile of ADSCs of each of the participants in this study. (DOCX 54 kb) [file 13287_2016_444_MOESM2_ESM.docx]

**Additional file 2: Table S2.**
